# A report on the clinical-pathological correlations of 788 gingival lesion

**DOI:** 10.4317/medoral.21845

**Published:** 2017-10-21

**Authors:** Alessio Gambino, Mario Carbone, Roberto Broccoletti, Paola Carcieri, Davide Conrotto, Marco Carrozzo, Paolo G. Arduino

**Affiliations:** 1 DDS, MSc, PhDs, Department of Surgical Sciences, Oral Medicine Section, Lingotto Dental School, University of Turin, Turin, Italy; 2 MD, DMD, Department of Surgical Sciences, Oral Medicine Section, Lingotto Dental School, University of Turin, Turin, Italy; 3 DDS, Department of Surgical Sciences, Oral Medicine Section, Lingotto Dental School, University of Turin, Turin, Italy; 4 DH Department of Surgical Sciences, Oral Medicine Section, Lingotto Dental School, University of Turin, Turin, Italy; 5 DDS, MSc, Department of Surgical Sciences, Oral Medicine Section, Lingotto Dental School, University of Turin, Turin, Italy; 6 MD, DMD, Department of Oral Medicine, School of Dental Sciences, University of Newcastle upon Tyne, Newcastle upon Tyne, UK

## Abstract

**Background:**

The diagnosis and treatment of a variety of non-plaque related gingival diseases have become an integrated aspect of everyday dentistry. The aim of this study was to analyse the relationship between clinical appearance and histopathological features of gingival lesions in a large Northern Italian population.

**Material and Methods:**

A retrospective study of 788 cases of gingival and alveolar mucosal biopsies was set up. Statistical analysis was performed by calculating the odds ratio and 95% confidence interval (C.I.), in order to assess the degree of association between the clinical parameters considered (primary lesions) and the single pathologies, statistically evaluated by Mantel-Haenszel tests. The correlation between clinical and histological diagnosis was classified as follow: 1) expected data (ED): provisional clinical diagnosis; 2) real data (RD): final histopathology diagnosis; 3) concordant data (CD): correspondence between the expected data and real data. The correlation was calculated as follow: CC (complete concordance) = CD x 100 / ED, this expressing the percentage in which the clinical and the histological diagnosis overlapped.

**Results:**

The most frequently observed and biopsied primary lesions resulted to be exophytic, followed by mucosal colour changes and finally by losses of substance. The statistically significant association between primary lesion and their manifestation in gingival pathologies was reported. Volume increases, for instance, were positively correlated to plasma cell epulis, pyogenic granuloma, fibrous reactive hyperplasia and hemangioma. Verrucous-papillary lesions were most often seen in verrucous carcinoma, verrucous leukoplakia and mild dysplasia. White lesion resulted to be related to leukoplakia or oral lichen planus. Red lesions resulted to be related only oral lichen planus. Erosive vesicle-bullous lesions were linked to disimmune pathologies. Ulcerative lesions were positively associated to oral squamous cell cancer. Finally, potentially malignant disorders have the most percentage high concordance. Among the malignant lesions, the correlation increased up to the squamous cell carcinoma and leukaemia.

**Conclusions:**

This article presented the frequency and the clinico-pathological concordance of all primary lesions and the histopathological diagnosis of gingival lesions. For every primary lesion, it is possible to correlate a specific histopathological diagnosis in a statistical manner. This can be a valuable aid for not specialist clinicians who daily observe mucosae and have the opportunity to intercept major diseases.

** Key words:**Gingival lesions; clinical appearance; histological analysis; clinico-pathological correlation.

## Introduction

The gingiva is usually touched by different type of lesions, non-neoplastic and neoplastic, the latter either benign or malignant (1). A correct differential diagnosis with gingivitis, and periodontitis, is the first stage for recognizing a non-plaque induced gingival disease. Physical examination of the clinical appearance is the following step. This is because different primary lesions can be detected within the soft oral tissues: lesions causing a loss of substance (erosions, ulcers), exuberant lesions (volume increases, localized or diffuse with surface normal or warty), and variations in color (white areas, red areas, white and red areas, dark areas) (2).

Tissue biopsy and histological examination however represent the golden standard in diagnostic oral pathology and they are used to confirm the clinical diagnosis (3). A large number of lesions may require a microscopic analysis, including neoplasms (characterized by progressive autonomous growth that can be either a benign or a malignant course), all the lesions suspected as potentially malignant, and surely non-neoplastic lesions (that are usually inflammatory or represent a reaction to some kind of irritation or low grade injury) (4).

Hence, it could be essential to study the accuracy level of the clinical diagnoses made by physicians against the final diagnosis obtained by histopathological examination. To date, a paucity of data is available on the assessment of the diagnostic agreement between the clinical and histopathological diagnosis of oral soft tissue lesions (5). Only limited studies on gingival lesions have considered large enough populations and up-to-date literature does not provide a valid report regarding the epidemiology of gingival lesions within the Italian population. We recently reported the histopathological and clinical appearance of a huge gingival sample, emphasizing the importance of proper histological characterization and differential diagnosis for dentists (1).

The aim of this study was to retrospectively analyse the relationship, and concordance, between elementary lesions, clinical and definitive diagnosis of gingival diseases in a Northern Italian population.

## Material and Methods

Case records of patients, who had been referred to the Oral Medicine Unit (CIR-Dental School, Turin, Italy) for the diagnosis and management of gingival lesions in the period ranging December 1996 to December 2016, were considered. The relevant retrospective data were extrapolated, including demographic information, age and gender, smoking habits, alcohol consumption, clinical aspect of the lesions and localisation. Since 1991, in our unit, all patients with gingival disorders have been also referred to undergo a dental panoramic radiograph, in order to detail possible central lesions; for this reason, radiological data were moreover collected before oral surgery.

The following inclusion criteria were accepted: 1) all age groups and both genders; 2) reports with comprehensive and adequate case histories; 3) more than one sample for a given patient, as long as biopsied at different times (1). The clinicians, who were involved in filling out the records, had a recognized postgraduate training and appropriate qualifications (A.G., P.G.A.).

Data regarding the histological type of each lesion were retrieved from the biopsy register.

According to the clinical description obtained from the files, and since 2001 from digital pictures, the observed lesions were divided and classified by 2 expert oral physician (M.C., P.G.A.) into 7 groups: a) lesions which implied a volume increase; b) verrucous-papillary lesions; c) white lesions (homogeneous and heterogeneous); d) red lesions; e) pigmented lesions; f) erosive lesions; and g) ulcerative lesions.

All data collected from each patient were analysed using descriptive statistics; continuous variables were expressed as mean ± SD (standard deviation). Odds ratios (ORs) and 95% confidence intervals (95%CIs) were obtained performing a multivariable logistic regression models (adjusted for age at diagnosis, smoking status and alcohol consumption), in order to assess the degree of association between the clinical parameters considered (primary lesions) and the single pathologies, statistically evaluated by Mantel-Haenszel tests. Values were considered significant at *P*<.05. All analyses were performed using SPSS® software (SPSS for windows, version 11, SPSS inc, Chicago, IL, USA). The correlation between clinical and histological diagnosis was classified as follow: 1) expected data (ED): provisional clinical diagnosis; 2) real data (RD): final histopathology diagnosis; 3) concordant data (CD): correspondence between the expected data and real data. The correlation was calculated as follow: CC (complete concordance) = CD x 100 / ED, this expressing the percentage in which the clinical and the histological diagnosis overlapped. This method was adapted from the one previously reported by Patel and co-workers (3).

## Results

The total number of biopsied samples resulted to be 788. The study group involved 520 females and 268 males, and the mean age at presentation was 57.5 years for men (SD ± 13.6) and 54.1 years for women (SD ± 12.8).

The involved gingival localizations were: maxillary gingiva 321 cases, mandibular gingiva 264 cases, maxillary alveolar mucosa 118 cases, mandibular alveolar mucosa 85 cases.

The most frequently observed and biopsied primary lesions resulted to be exophytic (volume increase and verrucous-papillary lesions, 45%), followed by mucosal colour changes (white lesions, red lesions and pigmented lesions, 39%) and finally by losses of substance (erosions and ulcers, 16%).

Amongst exophytic lesions, those characterized by a volume increase were the most commonly observed; of these, the largest number of biopsies was carried out on hyperplastic reactive fibrosis and peripheral giant cell granuloma. Verrucous-papillary lesions were also often found, and proliferative verrucous leukoplakia seemed to be the most common of these ( Table 1).

**Table 1:**
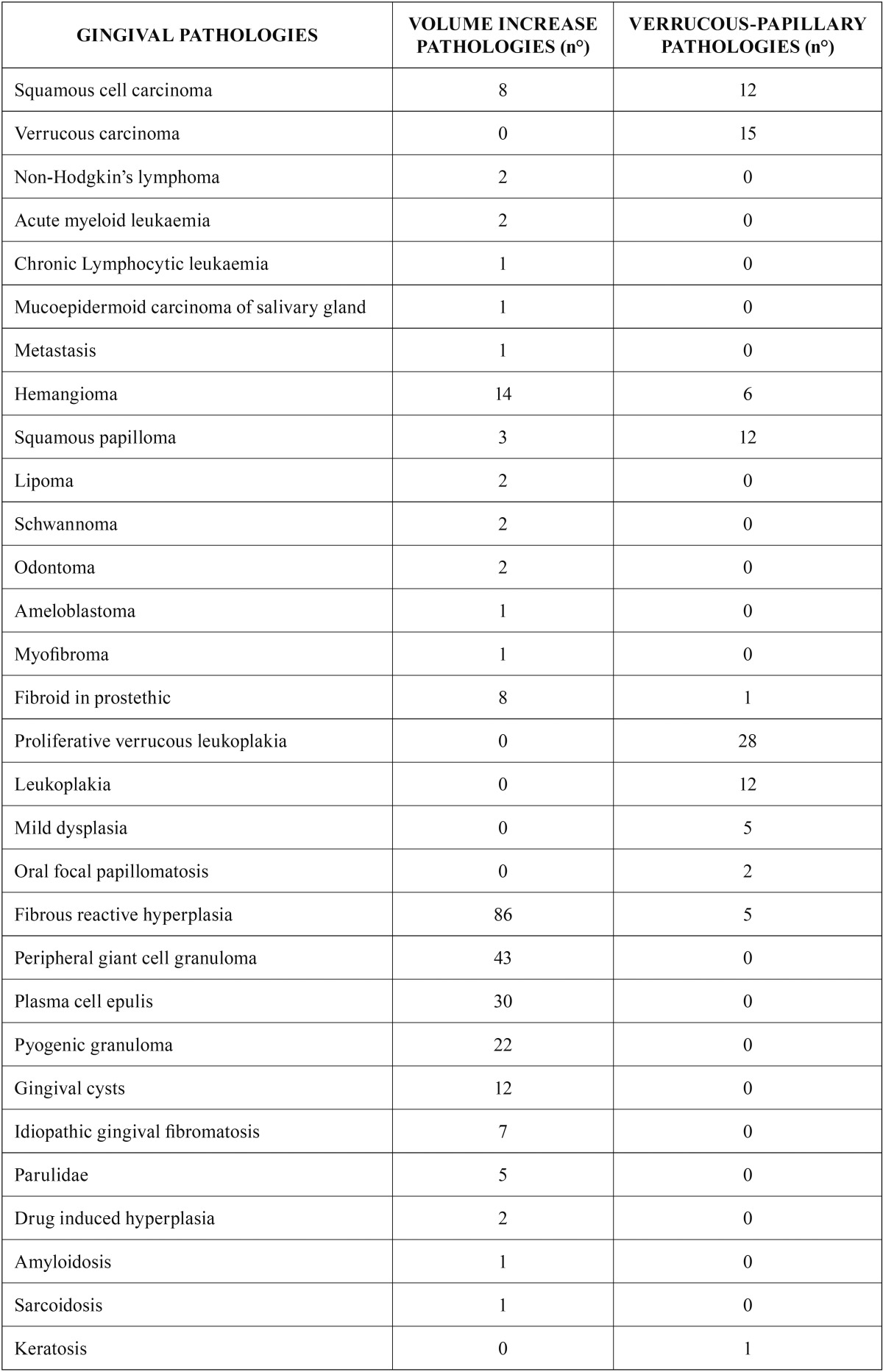
Described frequency of pathologies relate to exuberant lesions.

When considering colour changes, white lesions were the most frequently encountered (both homogeneous and non-homogeneous plaques and stains) and, amongst these, leukoplakia and oral lichen planus were the most commonly described.

Cases of mucous membrane pemphigoid were the most frequent among those lesions showing a loss of substance, while cases of carcinoma frequently presented as ulcerations ( Table 2).

**Table 2:**
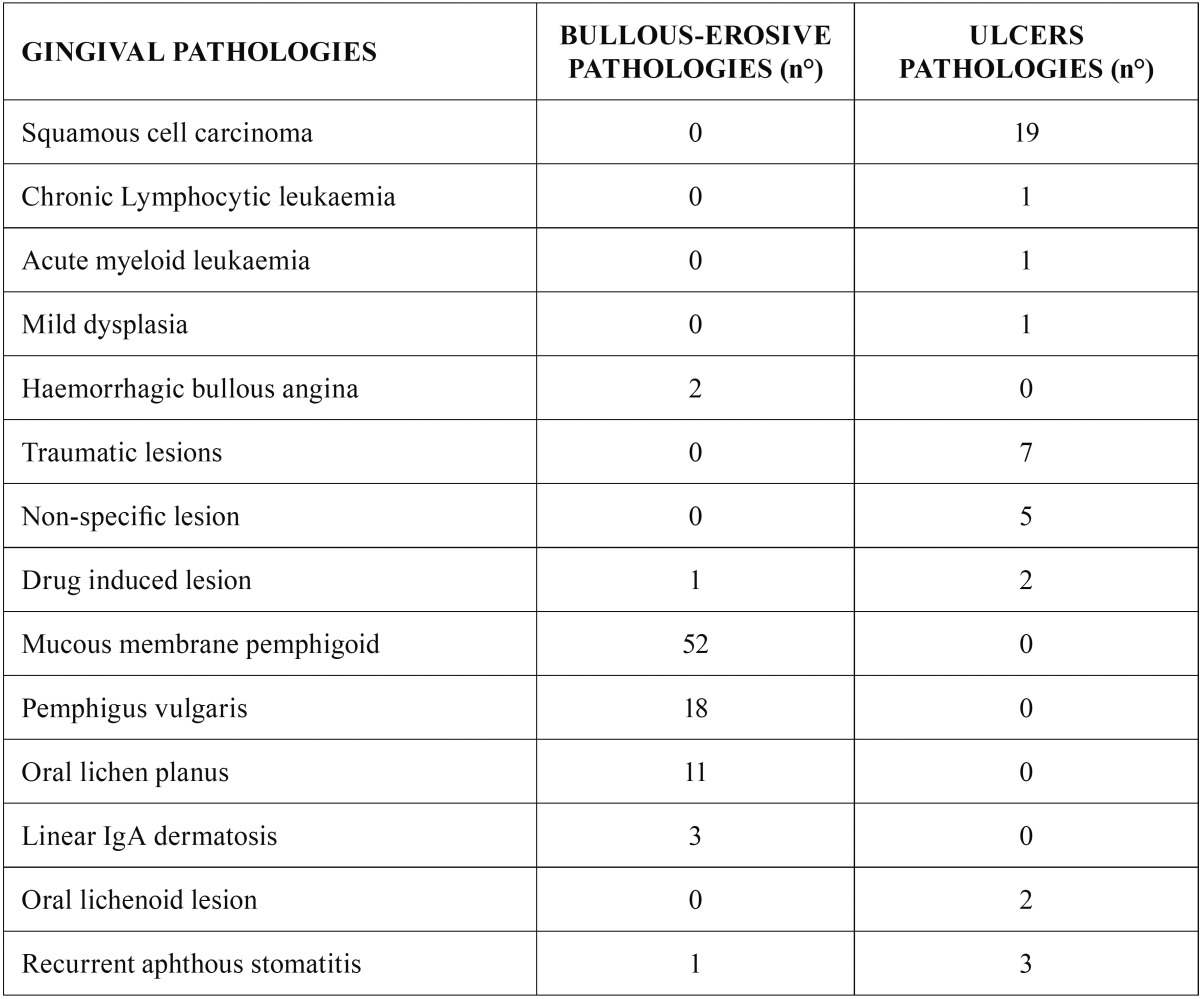
Described frequency of pathologies relate to loss of substance.

 Table 3 shows the statistically significant association between primary lesion and their manifestation in gingival pathologies. Volume increases, for instance, were positively correlated to plasma cell epulis, pyogenic granuloma, fibrous reactive hyperplasia and hemangioma (all benign neoplasms). Verrucous-papillary lesions were most often seen in verrucous carcinoma, verrucous leukoplakia and mild dysplasia. White lesion resulted to be related to leukoplakia or oral lichen planus. Red lesions resulted to be related only oral lichen planus. Erosive vesicle-bullous lesions were linked to disimmune pathologies. Ulcerative lesions were positively associated to oral squamous cell cancer.

**Table 3:**
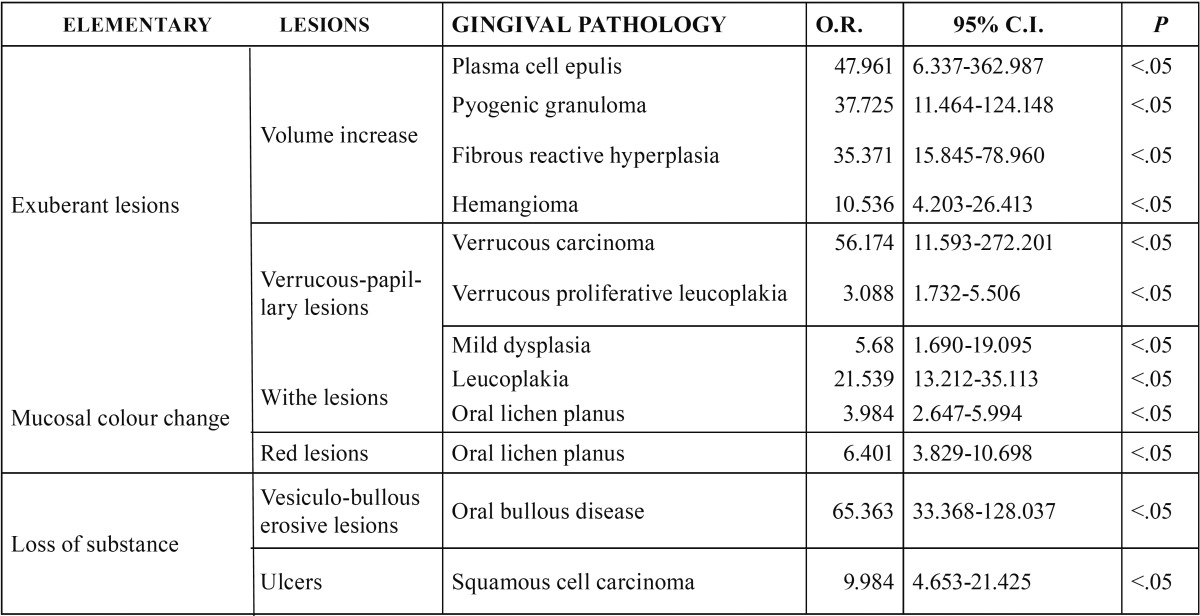
Statistical positive associations between elementary lesions and no-plaque induced gingival diseases.

**Figure 1:**
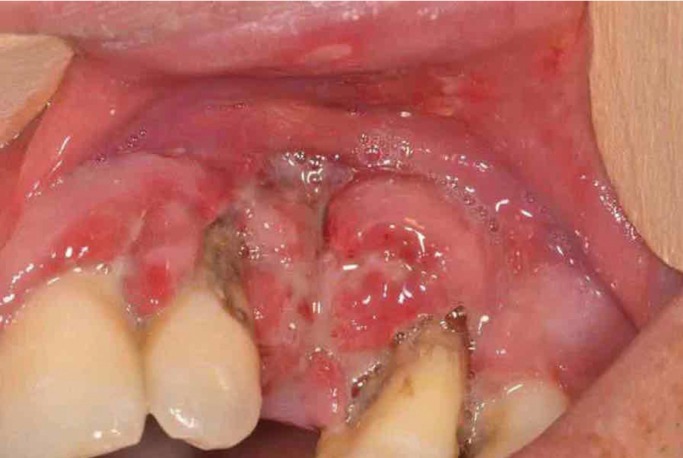
Squamous cell carcinoma on the maxillary gingival.

**Figure 2:**
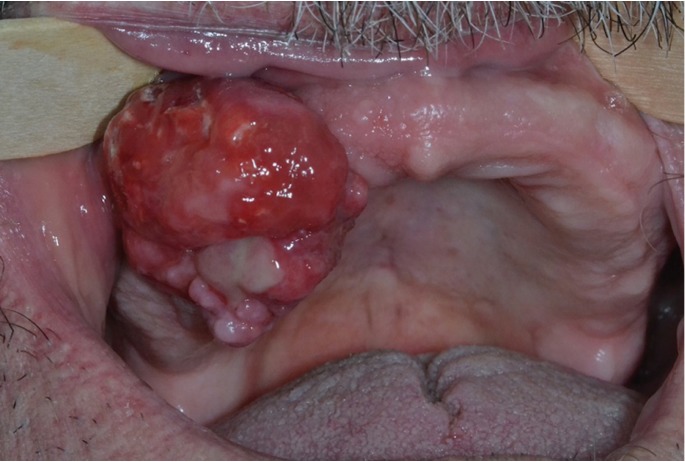
Giant cell granuloma on the maxillary edentulous ridge.

**Figure 3:**
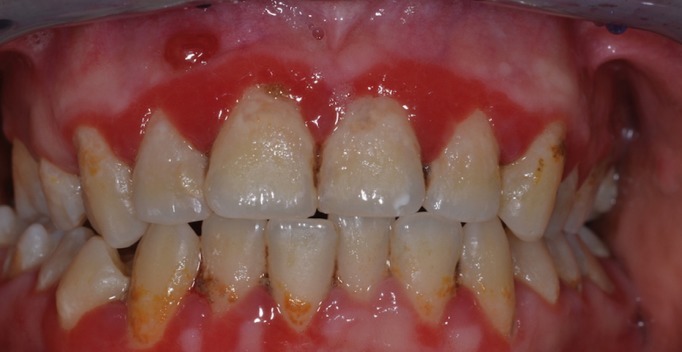
Gingival manifestation of mucous membrane pemphigoid.

 Table 4 shows the correlation between the clinical diagnostic hypothesis and the result of the histopathological examination. The gingival diseases were divided in four categories: malignant, potentially malignant, benign and disimmune. Potentially malignant disorders have the most percentage high concordance. Among the malignant lesions, the correlation increased up to the squamous cell carcinoma and leukaemia (Figs. 1-3).

**Table 4:**
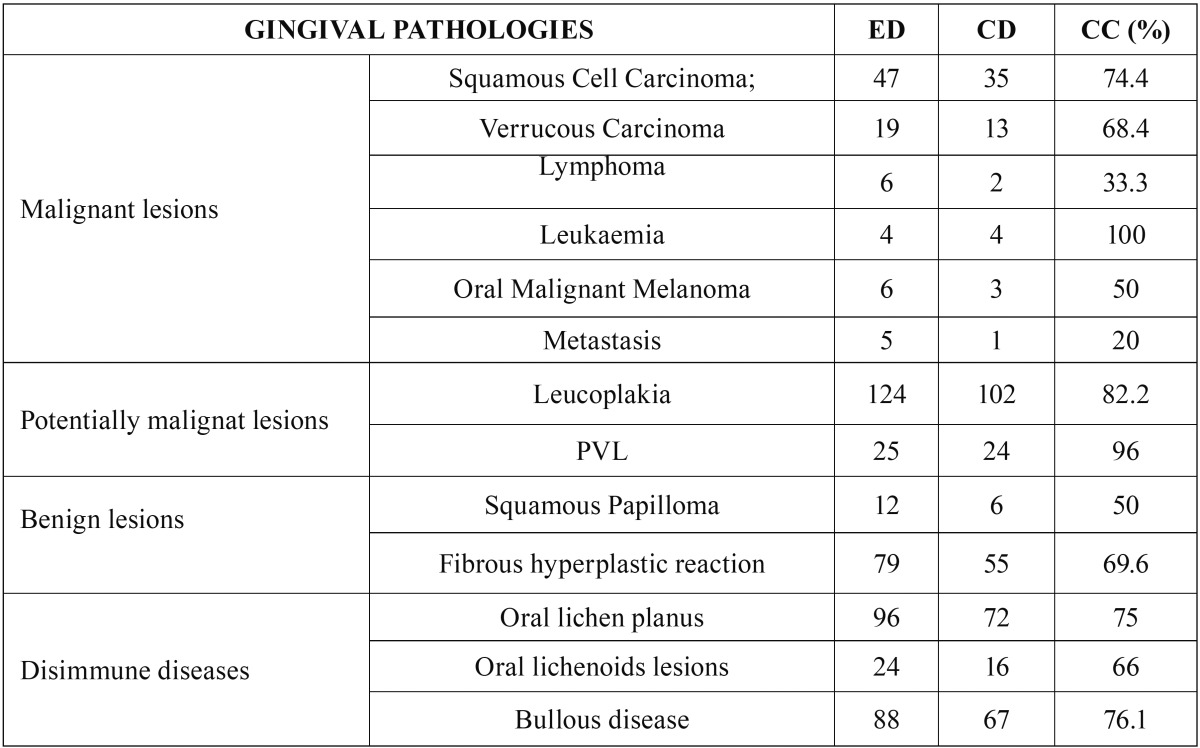
Correlation between clinical and histological diagnosis. The correlation between clinical and histological diagnosis was classified as follow: 1) expected data (ED): provisional clinical diagnosis; 2) real data (RD): final histopathology diagnosis; 3) concordant data (CD): correspondence between the expected data and real data. The correlation was calculated as follow: CC (complete concordance) = CD x 100 / ED, this expressing the percentage in which the clinical and the histological diagnosis overlapped.

## Discussion

To the best of our knowledge, this is the first study to consider the clinical-pathological correlation of non-plaque induced gingival lesions in an Italian population. Some of the cases of this report have been previously reported by our group (1).

The accurate diagnosis and treatment of pathological features of the gingiva should be a basic and fundamental aspect of everyday dentistry. The appearance of the lesion itself often provides valuable diagnostic information. Thus, many experienced clinicians use visual inspection and palpation to obtain an accurate provisional diagnosis (6). Many of the described lesions are non-plaque related if correlated to regular plaque-induced gingivitis and periodontitis.

The originality of this work is that of aiming at establishing a correlation between clinical and pathological findings in gingival lesions. In fact, a correlation between primary lesion and subsequent histological diagnosis, shows as some gingival disease non-plaque induced appear more frequently than others with a particular clinical aspect into a specific population. In agreement with previous similar studies (7-9), exophytic masses represented the largest group of lesions undergoing gingival biopsies, statistically related to benign neoplasms and non-neoplastic lesions. The hemangioma was the most frequent among benign neoplasms, and fibrous reactive hyperplasia was the most commonly biopsied lesion within the non-neoplastic group.

Verrucous papillary lesions in the gingiva and alveolar mucosa were strongly related to verrucous carcinoma. This data is already documented in the literature (10,11). Proliferative verrucous leukoplakia (PVL) is essentially clinically diagnosed retrospectively and has a high rate of malignant transformation (12). This entity war also frequently described as verrucous lesions mainly occurring in the gingiva.

White lesions resulted to be statistically correlated to leukoplakia, oral lichen planus and mild dysplasia. Oral lichen planus was also associated with red lesions, being the disimmune gingival pathology that most commonly caused gingival desquamation, as already showed by our group (1). Oral white lesions have gained much attention in cancer detection and control. Although most white lesions are histologically a benign hyperkeratosis, others with a similar clinical appearance have been associated with a continuum of features ranging from mild to severe dysplasia to actual carcinoma (13-15). White and red lesions are often biopsied because of their link to potentially malignant lesions. A positive association with leukoplakia or lichen planus being at risk of malignant progression and with epithelial dysplasia intended as “precursor” or lesion within which there is already a potential histological malignant transformation. In fact, dysplasia usually develops over a pre-existing white lesion homogeneous or inhomogeneous and warrants more aggressive management (16,17).

Ulcers are strictly bound to oral squamous cell carcinoma, representing 50% of all ulcerations within our study. Oral cancer, at and advance stage, usually manifests itself with ulcers and lumps with irregular margins which are rigid to touch. The different diagnosis should be established with other oral malignant diseases or traumatic lesions, that however are quite uncommon on the gingival tissues (18). Disimmune erosive diseases often present oral manifestations, with possible preference for the gingival tissues (19,20), characterized by an intense inflammatory reaction with clinical or subclinical thinning or vesicle formation and immediate ulceration, associated to severe lesional and perilesional reddening. Gingival lesions very often precede signs in other parts of the body, and in such situations, are referred to as heraldic manifestations (21,22).

This is in agreement with the present study: in fact, we have observed a high concordance between bullous erosive diseases and disimmune pathologies, like mucous membrane pemphigoid, and pemphigus vulgaris, with gingival manifestation.

A contemporary survey examining the current approaches to the diagnosis and management of oral premalignant lesions among the American Board of Specialists in Oral Medicine revealed that most of the clinicians act on an initial clinical diagnosis before embarking on a biopsy to establish a tissue diagnosis (23). This can be argued to be beneficial for beginning treatment without delay. However, if this approach is to be successful, the initial clinical diagnosis must be accurate and should not have missed any features.

Hence, it is important to study the accuracy level of the clinical diagnoses made by clinicians against the final diagnosis obtained by histopathological examination (24).

The association between primary lesion and subsequent histological findings reveals that some non-plaque induced gingival pathologies are more common than others and present specific clinical features. This can be of great help for a non-specialist who continuously examines gingival tissues. This is because the simple observation and description of the primary lesion represent the most important moment to formulate a diagnostic hypothesis and results to be extremely useful in the diagnostic process, which will end up in a specialist centre.

The main limitation of this study is that it is retrospective and the clinical data has been collected by different clinicians over different periods, therefore those data could be not representative; however, even if based on a long period, most of the clinical evaluation has been performed by the same clinicians who also trained the other during the proposed period.

According to Franklin and co-workers (24), this work could reflect an increasing demand of biopsy by general dentist for a diagnostic oral histopathology service and their use of this service should be encouraged, but dentists and dental hygienists must be trained to examine, diagnose, and manage oral diseases and arguably are the clinicians most familiar with the oral environment.

However, to validate these results, multicentre studies are needed to help and improve performance diagnostics of general dental practitioners and specialists.
